# Consumption of Milk and Alternatives and Their Contribution to Nutrient Intakes among Canadian Adults: Evidence from the 2015 Canadian Community Health Survey—Nutrition

**DOI:** 10.3390/nu11081948

**Published:** 2019-08-19

**Authors:** Olivia Auclair, Yang Han, Sergio A. Burgos

**Affiliations:** 1 Department of Animal Science, McGill University, Ste-Anne-de-Bellevue, QC H9X 3V9, Canada; 2 School of Human Nutrition, McGill University, Ste-Anne-de-Bellevue, QC H9X 3V9, Canada; 3 Department of Medicine, McGill University, Montréal, QC H4A 3J1, Canada; 4 Metabolic Disorders and Complications Program, Research Institute of McGill University Health Centre, Montréal, QC H4A 3J1, Canada

**Keywords:** dairy products, self-selected diets, nationally representative survey, Canada’s Food Guide, dietary intake

## Abstract

As a staple food and dense source of nutrients, milk and alternatives play an important role in nutrient adequacy. The aims of this study were to quantify the consumption of milk and alternatives within Canadian self-selected diets and determine their contribution to intakes of nutrients and energy. First, 24-h dietary recalls from the 2015 Canadian Community Health Survey—Nutrition were used to assess 1-d food and nutrient intakes among Canadian adults ≥19 y (*n* = 13,616). Foods were classified as milk and alternatives according to the 2007 Canada’s Food Guide. Descriptive statistics were used to calculate daily servings of milk and alternatives by different age groups and demographic characteristics. Population ratios were used to discern their contribution to total intakes of nutrients and energy. Mean daily servings (±SE) were highest for milk (0.60 ± 0.02) and cheese (0.42 ± 0.01), intermediate for frozen dairy (0.16 ± 0.01) and yoghurt (0.14 ± 0.01), and lowest for soy and other dairy (<0.03). Intakes were lowest among Canadians 51 + y (1.3 ± 0.03), females (1.25 ± 0.03), non-Caucasians (1.06 ± 0.05), those with less than a secondary education (1.19 ± 0.05), and British Columbians (1.17 ± 0.05). Milk and alternatives contributed >20% to total intakes of calcium (52.62 ± 0.46%), vitamin D (38.53 ± 0.78%), saturated fat (28.84 ± 0.51%), vitamin B12 (27.73 ± 0.57%), vitamin A (26.16 ± 0.58%), phosphorus (24.76 ± 0.35%), and riboflavin (24.43 ± 0.37%), of which milk was the top source. Milk and alternatives contribute substantially to nutrient intakes and thus warrant further attention in terms of mitigating nutrient inadequacy among the Canadian population.

## 1. Introduction

Dairy is a dense source of essential nutrients, ranging from vitamins and minerals to high-quality protein. However, dairy is often subject to scrutiny due to its saturated fat content, a nutrient that many national dietary guidelines, including those of the USA and Canada, recommend limiting due to its putative harmful association with cardiovascular health [1,2]. However, as a staple food within Canadian diets, it is important to quantify the consumption and contribution of dairy to nutrient intakes as a means of implementing proper policies to ensure the overall health of Canadians.

Consumption of dairy products in Canada is shifting. Over the past two decades, the intake of fluid milk has been declining concurrently with a rise in the consumption of solid dairy foods, such as cheese and yoghurt [3]. These changes can be attributed to the large proportion of elderly people in the Canadian population, a demographic known to consume fewer beverages such as milk [4,5]. Shifts in dairy consumption can also be caused by changing dietary preferences and the increasing popularity of plant-based alternatives, such as soy, coconut, and almond beverages [4,6].

Canada’s Food Guide (CFG) is a set of dietary guidelines intended to promote healthy eating among Canadians [1]. Published in 2007, *Eating Well with Canada’s Food Guide* (referred to here as the 2007 CFG) reflects a food intake pattern that accounts for nutrient adequacy based on the Dietary Reference Intakes (DRI), nutrition-related chronic diseases, and input from public consultations [7]. The 2007 CFG consisted of four food groups—vegetables and fruit, grain products, milk and alternatives, and meat and alternatives—and an ‘other foods’ group, each assigned a daily number of servings recommended based on age and sex. In January 2019, Health Canada released an updated version of CFG. The recommendations outlined in the new guide were informed by high-quality evidence, including that from systematic reviews, assessing relationships between food and health [1]. The new guide took major strides away from the previous one, dismissing the concept of food groups in place of a food guide snapshot illustrated by a plate containing vegetables and fruits, whole grains, and protein sources. The current CFG places a major emphasis on plant-based sources of protein, resulting in the overall lower prominence of milk and alternatives within the nation’s present dietary guidelines.

According to Health Canada, many Canadians were not meeting the recommendations outlined in the 2007 CFG [8]. As a result, the prevalence of nutrient inadequacy was high, particularly for calcium, magnesium, zinc, vitamin A, and vitamin C. Constituting one of the few food groups containing considerable amounts of a wide range of nutrients, milk and alternatives have the potential to mitigate inadequate intakes of nutrients of concern in Canada. The de-emphasising of milk and alternatives in the new CFG may have further implications in terms of nutrient adequacy for Canadians. Therefore, the aims of this study were to quantify the consumption of milk and alternatives within the self-selected diets of Canadian adults, as well as their contribution to total intakes of nutrients and energy based on data from the 2015 Canadian Community Health Survey (CCHS)—Nutrition.

## 2. Materials and Methods

### 2.1. The 2015 CCHS—Nutrition

The CCHS is a nationally representative cross-sectional survey that collects information regarding Canadians’ health status, health determinants, and utilization of the healthcare system [9]. Administered annually, the survey is a multistage clustered design to ensure the inclusion of a minimum number of respondents from each of the provinces, from both rural and urban dwellings, and from each age‒sex group corresponding to those in the DRIs. The 2015 CCHS—Nutrition constitutes the second of two nutrition-focused surveys, the first having been conducted in 2004 (CCHS, Cycle 2.2, Nutrition (2004), later renamed the 2004 CCHS—Nutrition). The 2015 CCHS—Nutrition consists of two components: (1) 24-h dietary recall and (2) health. The 24-h dietary recall component collects information pertaining to the foods and beverages consumed by respondents 24 h prior to the interview, from midnight to midnight. The automated multiple-pass method is a computer-assisted interviewing instrument aimed at helping respondents recollect and report their consumption. The health component gathers information pertaining to respondents’ weight and height, physical activity, chronic health conditions, sociodemographic characteristics, and supplement intake. The 2015 CCHS—Nutrition targeted Canadians ≥1 y of age residing within the provinces. Members of the Canadian forces, individuals living on reserves or Aboriginal settlements, and the institutionalized population were excluded from the survey.

For the purpose of this study, respondents below 19 y of age were excluded (*n* = 6,568; 32.06% of sample). Pregnant (*n* = 116; 0.57% of sample) and breastfeeding (*n* = 187; 0.91% of sample) women were excluded due to a lack of data with which to calculate their total energy expenditure (TEE) [10]. Data from the first 24-h dietary recalls were utilized as only 37% of respondents completed a second recall [9]. Nutrient intakes from supplements were excluded in the present analyses to obtain estimates of the percent contribution of nutrients and energy from food sources alone, although this may lead to underestimations of intakes for specific nutrients. The final sample size was 13,616. Access to the 2015 CCHS—Nutrition Master Files was granted by Statistics Canada (Project No. 18-SSH-MCG-5516). Population surveys conducted by Statistics Canada were granted ethical approval under the authority of the Statistics Act of Canada. According to Article 2.2 of the Tri-Council Policy Statement on Ethical Conduct for Research Involving Humans [11], research involving information that is legally accessible to the public is based on the presence of a legally designated custodian/steward that protects its privacy and proprietary interests, such as Statistics Canada, and so is exempt of institutional Research Ethics Board review. All analyses were conducted at the McGill-Concordia Laboratory of the Quebec Inter-University Centre for Social Statistics (QICSS).

### 2.2. Data File Structure

Files in the 2015 CCHS—Nutrition that were used in the present analyses include the health component, vitamin and mineral supplements, 24-h dietary recall (HS) file, food and ingredient details (FID) file, and CFG description (CFGD) file [12]. The HS file contains information pertaining to total nutrient intakes derived from food sources reported in the first 24-h dietary recalls, as well as socio-demographic variables and sample weights. The FID file contains the nutrient values for all items reported in the 24-h dietary recalls, including basic foods and recipe components. The CFGD file is a supporting file linking foods listed in the FID file to the 2007 CFG, which includes a variable referencing the CFG serving size for food items, standardized to grams (i.e., one food guide serving of 2% milk is equivalent to 250 mL or 257.819 g). The CCHS also contains a bootstrap weight file, each record containing 500 bootstrap weights, which are used to calculate confidence intervals around point estimates.

### 2.3. Food Classification

The Canadian Nutrient File (CNF) is Canada’s reference food composition database containing the nutrient profiles of over 5000 foods [9]. Data from the CNF and an accompanying recipe file, both from Health Canada, were used to assign food codes to items reported in the 24-h dietary recalls. Additionally, the CNF/CFG classification was developed by Health Canada as a surveillance tool to assess Canadians’ compliance with the 2007 CFG [13]. It constitutes a set of food codes that facilitates the classification of foods in the CNF into food groups and subgroups. Foods within the four core food groups were further classified into tiers based on how well they align with the guidance outlined in the 2007 CFG. The CNF/CFG classification divides milk and alternatives into two groups: (1) fluid milk and fortified soy-based beverages and (2) other milk alternatives (cheese, yoghurt). Despite being a plant-based alternative, Health Canada considered fortified soy-based beverages part of the milk and alternatives group as a high-calcium option for non-milk drinkers [14]. Some dairy products that are particularly high in fats, sugars, and/or sodium (i.e., butter and cream) are not considered within milk and alternatives according to the 2007 CFG and were therefore excluded from the analyses.

The Bureau of Nutritional Sciences (BNS) food codes were developed by Health Canada as a means of determining the contribution of select food categories to total nutrient intakes [12]. In this study, the BNS codes were utilized to classify foods within the CNF/CFG classification of milk and alternatives into sources, including milk, cheese, yoghurt, and frozen dairy. The Nutrition Survey System (NSS) food codes, as assigned uniquely to each food item, were used to identify soy products within the CNF/CFG classification of milk and alternatives, which consisted of fortified soy-based beverages as a standalone product or component of a product. Milk and alternatives that did not fall into these categories (i.e., cakes, milk-based beverages, soups, sauces, etc.) were classified as other dairy. The classification of milk and alternatives based on their respective food codes are listed in Appendix A.

### 2.4. Data Handling

Consumption of milk and alternatives was assessed by age and demographic characteristics in terms of the 2007 CFG servings. A new variable was created for food guide servings by dividing the food weight (g) by the Food Guide Serving (g). New variables were created to classify individuals into age groups according to those in the 2007 CFG: 19–50 years and 51+ years. New variables were also created for sex, level of education, household income, ethnicity, and province of residence.

As a self-reported dietary assessment tool, 24-h recalls are prone to bias due to misreporting. Energy intake tends to be under-reported, particularly in North America, Europe, and Australia [15]. Misreporting was detected based on a method outlined by Garriguet (2018). Respondents were classified as energy under-reporters, plausible-reporters, and over-reporters based on the ratio of their energy intake (EI) to TEE. The Institute of Medicine (IOM) equations were used to estimate TEE based on sex, age, height, weight, and physical activity [1]. Individuals were assumed to be sedentary based on Statistics Canada data regarding measured physical activity of adults from 2007 to 2015 [15]. The method of McCrory et al. [16] was used to classify respondents as under-reporters, plausible-reporters, or over-reporters if the percentage of their TEE that was reported as EI was <70%, 70–142%, or >142%, respectively. Only respondents with measured heights and weights were used in the IOM equations.

### 2.5. Statistical Analyses

Twenty-four-hour dietary recalls are not necessarily representative of an individual’s usual dietary intake, which varies from day to day. This within-person variation can lead to overestimations regarding the proportion of individuals with high or low intakes of a given food or nutrient [17]. The average of individuals’ 1-d intakes is, however, an appropriate estimate of the average usual intakes of a population [10]. Therefore, descriptive statistics were used to calculate intakes of milk and alternatives across age groups and demographic variables in order to assess compliance with the recommendations outlined in the 2007 CFG (two servings/d for 19–50 y and three servings/d for 51+ y, regardless of sex) [18].

The percentage contribution of nutrients and energy from milk and alternatives to total intakes were calculated as population ratios, which have been shown to provide better estimates of population usual intakes in contrast to other methods [19]. To calculate population ratios, nutrients deriving from milk and alternatives were summed across all individuals and divided by the sum of total intakes of that nutrient for all individuals, as done previously by Kirkpatrick et al. [19]. Ratios were used to rank nutrients and energy from milk and alternatives based on their percentage contribution to total intakes. Similarly, milk and alternative sources were ranked according to their percentage contribution to total intakes of nutrients and energy. The ranking of milk and alternative sources is attributable to how often the source is consumed and the quantity of the nutrient present in the source [19]. In order to aid in the interpretation of the ranking of milk and alternative sources, cross-tabulations were employed to determine the proportion of individuals who reported consuming milk and alternatives. Consumers were identified as individuals who reported >0 g of milk and alternatives in their 24-h dietary recall. Descriptive statistics were further utilized to discern 1-d intakes of nutrients and energy from milk and alternatives per capita and per consumer.

Weighting was used to obtain representative estimates for the Canadian population. As calculated by Statistics Canada, sample weights are assigned to each respondent and correspond to the number of individuals within the Canadian population represented by that respondent. To account for the complex multistage sampling frame of the 2015 CCHS—Nutrition, variance estimation was performed using the bootstrap balanced repeated replication technique [20,21]. All statistical analyses were performed using SAS (version 9.3; SAS Institute Inc., Cary, NC, USA) and SAS-callable SUDAAN software. Alpha was set at 0.05 for all statistical tests.

## 3. Results

### 3.1. Sample Characteristics

As shown in Table 1, the sample was split evenly among males and females. The majority of surveyed individuals were 19–50 y of age, Caucasian, had some post-secondary education, a household income less than CAD$50,000/y, resided within the province of Ontario, and were plausible energy reporters.

### 3.2. Consumption of Milk and Alternatives by Age and Demographics

Consumption of milk and alternatives among age groups and demographic variables are presented in Table 2. Daily servings of milk and alternatives averaged 1.36 ± 0.03. Consumption of milk and cheese constituted ~44% and ~30% of mean total intakes, whereas yoghurt and frozen dairy each made up ~10%, respectively. Other dairy and soy products were consumed in negligible amounts (<3% each) and thus are not reported in the tables. Exclusion of fortified soy-based beverages from milk and alternatives altered the estimates to such a small degree that the overall conclusions remained unchanged.

Overall, the 19–50 y age group consumed more daily servings of milk and alternatives compared to the 51+ y group (+0.12 servings/d; *p* = 0.02). Specifically, those 19–50 y had higher intakes of milk (+0.07 servings/d; *p* = 0.04) and cheese (+0.12 servings/d; *p* < 0.0001) compared to those 51+ y. Consumption of yoghurt and frozen dairy did not differ among age groups.

Males had higher intakes of milk and alternatives compared to females (+0.22 servings/d; *p* < 0.0001). Consumption of milk and cheese was also higher for males (+0.12 and +0.15 servings/d, respectively; *p* < 0.0001), whereas consumption of yoghurt was higher for females (+0.05 servings/d; *p* = 0.0002). Similar results were obtained among sexes within the 19–50 y and 51+ y age groups.

Caucasians had higher intakes of milk and alternatives compared to non-Caucasians (+0.41 servings/d; *p* < 0.0001). Consumption among Caucasians was higher for milk (+0.12; *p* = 0.001), cheese (+0.21; *p* < 0.0001), and yoghurt (+0.03; *p* = 0.0002). Similar observations were obtained across age groups, with the exception of Caucasians 19–50 y, whose consumption did not differ from that of non-Caucasians. Consumption of frozen dairy did not differ among ethnicities except in the 51+ y age group, for which Caucasians had higher intakes than non-Caucasians (+0.09 servings/d; *p* = 0.002).

The less than secondary education level was used as a reference with which to make comparisons regarding the consumption of milk and alternatives among age groups and education levels. Respondents with less than secondary education had lower intakes of milk and alternatives compared to those with some post-secondary (−0.26 servings/d; *p* = 0.0005) and post-secondary education (−0.19 servings/d; *p* = 0.005). The same pattern was observed in the 19–50 y age group, but not in the 51+ y group, for which consumption did not differ by education. Similarly, daily servings of milk differed among those 19–50 y; consumption was lower for those with less than secondary education compared to those with secondary (−0.15 servings/d; *p* = 0.03), some post-secondary (−0.21 servings/d; *p* = 0.01), and post-secondary education (−0.16 servings/d; *p* = 0.02). Cheese intake was higher among those with less than secondary compared to those with some post-secondary education (−0.12 servings/d; *p* = 0.003), yet differences were not observed among age groups. Consumption of yoghurt was also lower for those with less than secondary education compared to all other levels (−0.06, −0.08, and −0.11, respectively; *p* < 0.0001), a similar pattern of which was observed among all age groups. Finally, daily servings of frozen dairy did not differ among education levels except in the 19–50 y age group, consumption of which was lower for those with secondary compared to post-secondary education (−0.08 servings/d; *p* = 0.04).

Total daily servings of milk and alternatives did not differ among income levels, nor did they differ among age groups. Cheese consumption, however, was lower for households with <CAD$50,000/y compared to those with CAD$100,000–150,000/y (−0.08 servings/d; *p* = 0.03). Alternatively, daily servings of milk were higher for households with <CAD$50,000/y compared to those with CAD$50,000–100,000/y (+0.08 servings/d; *p* = 0.03). Consumption of yoghurt and frozen dairy did not differ among income levels.

British Columbia was used as a reference by which to draw comparisons among consumption of milk and alternatives among age groups and provinces. Residents of British Columbia consumed fewer daily servings of milk and alternatives compared to those of Quebec (−0.37; *p* < 0.0001), Alberta (−0.26; *p* = 0.001), Manitoba (−0.26; *p* = 0.01), Saskatchewan (−0.23; *p* = 0.02), and the Atlantic provinces (−0.19; *p* = 0.003). Daily servings of milk and alternatives were also lowest for British Columbians among all age groups. In particular, the consumption of milk was lowest for residents of British Columbia compared to all other provinces. Cheese intake was also lower for residents of British Columbia compared to Quebec (−0.16; *p* = 0.0003) and Alberta (−0.15; *p* = 0.002); the same differences were observed in the 19–50 y age group, whereas consumption only differed with respect to Quebec in the 51+ y group. Compared to British Columbians, daily servings of yoghurt were higher among residents of Quebec (+0.06; *p* = 0.007) and lower among residents of Ontario (−0.03; *p* = 0.03). In the 19–50 y age group, British Columbians also had lower intakes compared to Quebec (−0.1; *p* = 0.002), whereas in the 51+ y group, residents had higher intakes compared to those of Saskatchewan (+0.07; *p* = 0.007) and Ontario (−0.06; *p* = 0.009). No differences were observed for intakes of frozen dairy among age groups and provinces.

Intakes of milk and alternatives among energy reporters are also reported in Table 2. Generally, intakes were lowest for under-reporters, intermediate for plausible-reporters, and highest for over-reporters. Total consumption of milk and alternatives, as well as cheese and yoghurt, was higher for plausible-reporters compared to under-reporters, regardless of age. Daily servings of frozen dairy for plausible-reporters in the 19–50 y group, however, did not differ from that of under-reporters.

### 3.3. Contribution of Nutrients and Energy from Milk and Alternatives to Total Nutrient Intakes

The percentage contribution of nutrients and energy from milk and alternatives to total intakes and their per capita mean 1-d intakes are in Table 3. Nutrients from milk and alternatives contributing more than 20% to total intakes included calcium, vitamin D, saturated fat, vitamin B12, vitamin A, phosphorus, and riboflavin. Between 10% and 20% of Canadian adults’ total intakes of protein, sugar, zinc, total fat, cholesterol, potassium, sodium, monounsaturated fat, and energy were obtained from milk and alternatives. Finally, milk and alternatives contributed less than 10% to total intakes of magnesium, niacin, carbohydrates, linolenic acid, vitamin B6, thiamine, folate, polyunsaturated fat, linoleic acid, iron, fibre, and vitamin C.

The percentage contribution of nutrients and energy from milk and alternative sources to total intakes, as well as the per capita and per consumer mean 1-d intakes, are in Table 4. On any given day, 91.49 ± 0.51% of Canadian adults reported consuming milk and alternatives. Consumption was highest for milk (76.45 ± 0.75%) and cheese (54.74 ± 0.87%), intermediate for yoghurt (19.06 ± 0.72%) and frozen dairy (11.06 ± 0.58%), and lowest for other dairy (2.78 ± 0.22%) and soy (1.67 ± 0.21%). Milk and cheese ranked as the top milk and alternative sources contributing to total nutrient intakes. Milk was the primary contributor among all nutrients, contributing >20% to total intakes of sugar, potassium, magnesium, carbohydrates, thiamine, vitamin B6, folate, and vitamin C. Cheese was the top contributor for all remaining nutrients, as well as energy. Frozen dairy was the top contributor of fibre, but in negligible amounts (<1%).

## 4. Discussion

The present study characterizes the consumption of milk and alternatives among different age groups and demographic variables, as well as their contribution to total intakes of nutrients and energy. Milk and alternatives are staples within Canadian diets, providing many nutrients relative to their calories. Consequently, the de-emphasis of milk and alternatives in the current CFG may have negative consequences in terms of nutrient adequacy if Canadians fail to replace dairy with other nutrient-dense foods. Even so, the quantity and quality of nutrients from plant sources vary from those of milk and alternatives. It is therefore imperative that Canadians are made aware of the nutritional trade-offs regarding the replacement of milk and alternatives within habitual diets.

Canadian adults did not meet the recommended daily servings for milk and alternatives, regardless of the stratification method. Mean intakes of milk and alternatives among Canadian adults averaged 1.36 ± 0.03 servings/d. Based on data from the 2004 CCHS—Nutrition, the Evidence Review Cycle for Dietary Guidance (ERC) 2015 is a technical report commissioned by Health Canada to review the evidence underpinning dietary guidance [8]. Findings from the ERC 2015 revealed that more than half of Canadian adults did not meet the recommended number of servings for milk and alternatives outlined in the 2007 CFG and that daily servings of milk did not exceed two for any of the age‒sex groups. This study also found that adults 51+ y consume fewer servings of milk and alternatives compared to those 19–50 y, despite the higher number of servings recommended. Averaging 1.6 servings/d for all energy reporters and ages combined, Tugault-Lafleur and Black (2019) also reported intake of milk and alternatives as decreasing with age. With milk as the most commonly consumed dairy product with the highest average daily servings, the lower mean tabulated for adults is likely due to the association between decreasing beverage consumption and increasing age [5]. Low intakes of milk and alternatives have various health implications, particularly with regards to the maintenance of bone and muscle mass in the elderly population [22,23].

Health Canada considers fortified soy-based beverages part of milk and alternatives, while excluding dairy products that are particularly high in fat (i.e., butter and cream). Therefore, while a direct comparison can be made with other studies assessing the intake of milk and alternatives, comparing with those on dairy intake must be done with caution. Like the 2007 CFG, the 2015–2020 Dietary Guidelines for Americans (DGA) recommends fortified soy-based beverages as a component of their dairy food group [2]. There are some apparent differences in dairy consumption trends in the USA and Canada; for one, dairy consumption in the USA is higher than in Canada. The consumption is approximately 1.7 servings/d for adults 19–50 y, which decreases to 1.3 servings/d for those above 70 y of age [24]. Among individuals 19–50 y, consumption of milk and cheese is the same (0.8 servings/d); whereas cheese consumption decreases with age, milk intake increases to 0.9 servings/d in the 71+ y age group. Yoghurt consumption is lower than that of Canadians at 0.1 servings/d. Furthermore, like Canadians, approximately 99% of American adults do not meet the 2.5–3 servings/d recommendation for dairy outlined in the DGA.

Consumption of milk and alternatives varied among demographic variables. Caucasians consumed more daily servings of milk and alternatives compared to non-Caucasians. The higher daily servings of milk and alternatives amongst Caucasians could be attributed to their proportion in the Canadian population, constituting >70% of the study sample. Additionally, other studies have found that African Americans and other ethnic minorities consume less milk and alternatives compared to Caucasians [25,26]. The high incidence of lactose intolerance and lactase nonpersistence, as well as varying cultural preferences among ethnic minorities, may play a role in the lower consumption of dairy among such groups [27,28]. Canadians with less than secondary education consumed fewer daily servings of milk and alternatives compared to other education levels. Furthermore, the consumption of milk and alternatives did not differ among household income levels apart from milk and cheese, the consumption of which was lower in the <CAD$50,000/y income group. Associations between the consumption of milk and alternatives and variables such as education and income are inconsistent [29,30]. Darmon and Drewnowski (2008) reported no difference in the consumption of milk and alternatives among individuals of low and high socioeconomic status (education and income being indices of socioeconomic status), with the exception of cheese, which was consumed in greater amounts among individuals of high socioeconomic status [31]. Moreover, Kirkpatrick and Tarasuk [32] found that lower-income households consume fewer servings of dairy compared to higher income households, despite allocating a greater proportion of their spending towards milk and alternatives.

Nutrients from milk and alternatives contributed >20% to total intakes for calcium, vitamin D, saturated fat, vitamin B12, vitamin A, phosphorus, and riboflavin, among which milk was the top contributor. The contribution of milk and alternatives to nutrient intakes among Canadian adults is similar to those observed in other countries. In the USA, dairy contributed 47% to total calcium intakes, 42% to retinol, and 65% to vitamin D [33]. Among French adults, dairy consumption contributed significantly (>25%) to intakes of calcium, iodine, and riboflavin and moderately (between 10% and 25%) to intakes of phosphorus, zinc, retinol, and vitamin D [34]. In the Netherlands, the contribution of dairy to nutrient intakes was highest for calcium, vitamin B12, zinc, selenium, and folic acid and lowest for vitamin D, vitamin C, copper, and iron among adults [35]. Nevertheless, there is a high prevalence of nutrient inadequacy among the Canadian population. As evidenced from the ERC 2015, Canadian adults had inadequate intakes of calcium, magnesium, zinc, vitamin A, and vitamin C, most of which are abundantly present in milk and alternatives [8].

Approximately half of Canadian adults’ daily calcium intakes are derived from milk and alternatives. Evidence from the ERC 2015 confirms milk and alternatives as the most significant sources of calcium for Canadians [8]. However, the prevalence of calcium inadequacy in Canada ranged from 23% to 97% depending on age and sex, being highest among women >50 y and men >71. Calcium is an essential nutrient, particularly with regards to bone health. In the long term, deficiency caused by inadequate dietary intake or poor absorption can diminish bone mass and lead to osteoporosis [36]. Osteoporosis is one of the top nutrition-related chronic diseases in Canada, affecting 10% of the population above the age of 40, the prevalence of which is four times higher in women [8]. Postmenopausal women are particularly at risk of calcium deficiency due to decreased levels of oestrogen, which increases bone resorption [37]. Osteoporosis leads to increases in bone fracture risk, which is associated with higher overall morbidity and mortality [38]. Milk and alternatives were also prominent contributors to intakes of vitamin D (approximately 38%). Despite 75% to 96% of Canadians having inadequate intakes, blood measures did not point to widespread deficiency [8]. The mandatory fortification of fluid milk with vitamin D, implemented in the *Food and Drugs Act* of 1975, led to the elimination of rickets in children [39]. The ERC 2015 revealed that the majority of Canadians obtain vitamin D through fortified foods such as milk; however, the contribution of vitamin D from milk and alternative sources was found to decrease with age. Calcium and vitamin D work synergistically, so inadequate intakes can lead to reduced calcium absorption and loss of bone mass [40,41]. Since milk and alternatives are primary contributors of calcium and vitamin D, their lesser prominence within the current dietary guidelines may have further repercussions for the intakes of these two nutrients.

The contribution of milk and alternatives to magnesium intakes among Canadian adults was low (approximately 5%). In Canada, >10% of males and females >14 y had inadequate intakes of magnesium [8]. Magnesium is an essential mineral that functions as a cofactor in a number of enzymatic reactions [36]. Evidence from epidemiological studies points to an inverse association between magnesium intake and cardiovascular disease [42]. Although rare among healthy individuals, long-term magnesium deficiency is associated with hypocalcaemia and hypokalaemia, characterized by low calcium and potassium levels, respectively, thus exacerbating deficiencies of these nutrients [43,44].

Milk and alternatives contributed approximately 15% to Canadians’ total zinc intakes. However, more than 10% of males >30 y and females 9–50 and >70 y had inadequate intakes of zinc [8]. Zinc is an essential trace mineral and antioxidant involved in cell proliferation, reproduction, and immune function [45]. Deficiency is most common in developing countries, imparting harmful effects on pregnancy, susceptibility to infection, and neurobehavioral development [46,47]. Zinc can be obtained from dietary sources other than milk and alternatives, including oysters, red meat, poultry, beans, and nuts, but is less bioavailable from plant-based foods due to the presence of anti-nutritional factors that decrease its absorption in the small intestine [48]. Therefore, milk and alternatives are important sources of zinc, which is provided in abundance and in a bioavailable form.

The provision of vitamin A from milk and alternatives approximated 26%. The fortification of milk and butter substitutes with vitamin A, mandatory under Canada’s *Food and Drugs Act*, aids in providing Canadians with sufficient amounts of the vitamin to prevent deficiency [49]. However, the prevalence of vitamin A inadequacy was >10% for the Canadian population >9 y and highest for elderly men >70 [8]. Although rare in high-income countries, severe vitamin A deficiency leads to xerophthalmia, a disease characterized by blindness, impaired growth, and increased morbidity and mortality [50,51]. Preformed vitamin A from animal sources, including fortified milk and eggs, is more readily bioavailable than provitamin A carotenoids from plant sources [52]. As a matter of both quantity and quality, current recommendations towards primarily plant-based diets may have implications for vitamin A adequacy.

In addition to micronutrients, milk and alternatives are sources of high-quality protein. Animal sources, including dairy, are deemed of high quality due to their provision of all nine essential amino acids in forms that are readily available to the body [53,54]. In contrast, plant sources of protein are typically lacking in one or more essential amino acids and are therefore considered of lesser quality. The digestible indispensable amino acid score (DIAAS) is a measure of protein quality that uses the ileal digestibility of amino acids to determine their bioavailability [55]. Animal proteins are known to have a higher standard ileal digestibility than plant proteins. With a DIAAS ≥100, dairy proteins are considered excellent/high-quality sources compared to most other foods. Intake of high-quality protein supports muscle protein synthesis and the maintenance of muscle mass [56]. These attributes are most beneficial for the elderly population, a demographic at risk of sarcopenia, a disease characterized by progressive loss of muscle mass that leads to impaired physical function, frailty, and mortality [57]. Inadequate protein intake is one of the risk factors for sarcopenia. Thus, consumption of high-quality protein, particularly from dairy, is an important nutritional intervention strategy to promote muscle health [58].

Among Canadian adults, one-tenth of total energy intake was attributed to milk and alternatives. Relative to their calories, milk and alternatives are nutrient-dense food sources, providing a range of vitamins, minerals, and high-quality protein in compact form [59]. In contrast, energy-dense foods are those that contain a high amount of calories relative to their nutritional content. One study from the US found that the top dietary sources of calories were also some of the top contributors of nutrients, particularly among those of concern [60]. Therefore, the trade-offs between energy and nutrients as provided by various food sources are important considerations towards their inclusion in a healthful diet.

Milk and alternatives provide a range of essential nutrients, but are also sources of nutrients to limit. Saturated fat was the third most abundant nutrient contributed by milk and alternatives (approximately one-third of total intakes), to which cheese was the largest contributor. Kirkpatrick et al. (2019) reported milk as the second highest contributor of saturated fat in Canadian self-selected diets, behind cheese, although they accounted for the consumption of respondents ≥1 y of age [19]. Excessive intakes of saturated fat are associated with nutrition-related chronic diseases including cardiovascular disease and type 2 diabetes [61,62]. Nonetheless, nutrition research is shifting focus away from single nutrients and towards whole foods [63]. Despite the high concentration of saturated fat inherent to dairy, epidemiological studies have not shown negative associations regarding the consumption of regular-fat dairy products and cardiovascular-related clinical outcomes [64,65]. In addition to saturated fat, the current dietary recommendations outlined in the new CFG encourage limiting the intake of added sugars. Excessive sugar intakes are associated with a range of noncommunicable diseases, including obesity, cardiovascular disease, and dental caries [66]. As opposed to naturally occurring sugars such as lactose in unsweetened milk, dietary guidelines pinpoint added and free sugars as those to limit because they tend to be present in foods with few nutrients [67,68]. Although the CNF does not distinguish between naturally occurring and added sugars, the CNF/CFG classification set thresholds to detect the presence of added sugars in flavoured milks and yoghurts; foods with sugar contents beyond such thresholds were not considered within milk and alternatives according to the 2007 CFG [13]. This distinction between total, added, and free sugars warrants attention when assessing the contribution of milk and alternatives to sugar intake by Canadians.

There are several strengths to our study, including the use of the 2015 CCHS—Nutrition as a nationally representative survey. Furthermore, there were few missing values for nutrients due to the extensive nature of the CNF, which is 100% complete for macronutrients and energy and >95% complete for vitamins and minerals. However, there are some limitations to our study. Firstly, 1-d food and nutrient intakes collected from 24-h dietary recalls are not necessarily representative of usual dietary intakes. Distributions of usual intake would allow for estimates of the prevalence of Canadians below the recommendations for milk and alternatives outlined in the 2007 CFG [9]. Secondly, although energy misreporting was detected using previously validated methods, no adjustments were employed to correct for under- and over- reporting. However, as stated by Garriguet (2018), energy misreporting is a minimal source of bias in the 2015 CCHS—Nutrition. Thirdly, nutrient profiles from the CNF database are not complete for all foods reported in the 2015 CCHS—Nutrition [9]. As a result, several essential nutrients, including chromium, fluoride, iodine, and molybdenum, are not accounted for in this study.

## 5. Conclusions

Milk and alternatives are commonly consumed within the Canadian diet and provide a range of essential nutrients. However, Canadians are not meeting the recommendations for milk and alternatives outlined in the 2007 CFG. Intakes of milk and alternatives were found to be lowest among Canadians 51+ y and females, demographics that may benefit the most from such rich sources of calcium and high-quality protein. With the new food guide in place, the de-emphasis of animal-based foods such as dairy may compromise nutrient adequacy if not carefully replaced by nutrient-dense, plant-based protein sources. Therefore, consumers and policy-makers must take caution to ensure nutrient requirements are met, especially for those of concern, in the face of new recommendations to moderate the consumption of animal-based food sources. This research provides a baseline with which to compare future consumption of milk and alternatives and the top dietary sources contributing to the provision of nutrients and energy with the new food guide in place.

## Figures and Tables

**Table 1:** Demographic characteristics of the study sample from the 2015 Canadian Community Health Survey—Nutrition (*n* = 13,616).

Demographic Variable	Percentage (%) ±SE
Age Group	
19–50 y	54.31 ± 0.09
51+ y	45.69 ± 0.09
Sex	
Male	49.99 ± 0.10
Female	50.01 ± 0.10
Ethnicity	
Caucasian	73.69 ± 0.94
Non-Caucasian	26.31 ± 0.94
Education	
Less than secondary	12.34 ± 0.49
Secondary	25.96 ± 0.76
Some post-secondary	34.00 ± 0.83
Post-secondary	27.70 ± 0.86
Income (CAD$/y)	
<50,000	34.19 ± 0.85
50,000–100,000	32.42 ± 0.82
100,000–150,000	19.67 ± 0.75
>150,000	13.73 ± 0.68
Province	
British Columbia	13.36 ± 0.05
Alberta	11.31 ± 0.07
Saskatchewan	2.95 ± 0.01
Manitoba	3.33 ± 0.02
Ontario	38.72 ± 0.11
Quebec	23.58 ± 0.07
Atlantic provinces	6.75 ± 0.03
Reporter Status	
Plausible-reporters	59.35 ± 0.99
Under-reporters	32.21 ± 0.96
Over-reporters	8.44 ± 0.54

**Table 2:** Daily servings of milk and alternatives for all energy reporters by age groups and demographics characteristics based on mean 1-d intakes from the 2015 Canadian Community Health Survey—Nutrition (*n* = 13,616).

Variable	Milk and Alternatives		Milk		Cheese		Yoghurt		Frozen Dairy	
	19–50 y	51+ y	19–50 y	51+ y	19–50 y	51+ y	19–50 y	51+ y	19–50 y	51+ y
	Mean ± SE	Mean ± SE	Mean ± SE	Mean ± SE	Mean ± SE	Mean ± SE	Mean ± SE	Mean ± SE	Mean ± SE	Mean ± SE
Sex										
Males	1.57 ± 0.06 ^a^	1.36 ± 0.04 ^a^	0.69 ± 0.04 ^a^	0.62 ± 0.03 ^a^	0.56 ± 0.04 ^a^	0.4 ± 0.03 ^a^	0.12 ± 0.01 ^a^	0.12 ± 0.01 ^a^	0.17 ± 0.02	0.18 ± 0.02
Females	1.26 ± 0.05	1.24 ± 0.03	0.56 ± 0.03	0.51 ± 0.02	0.37 ± 0.02	0.31 ± 0.01	0.17 ± 0.02	0.17 ± 0.01	0.12 ± 0.02	0.19 ± 0.02
Ethnicity										
Caucasian	1.56 ± 0.05 ^b^	1.38 ± 0.03 ^b^	0.68 ± 0.03 ^b^	0.59 ± 0.02 ^b^	0.56 ± 0.03 ^b^	0.39 ± 0.02 ^b^	0.15 ± 0.01	0.16 ± 0.01 ^b^	0.14 ± 0.01	0.2 ± 0.02 ^b^
Non-Caucasian	1.14 ± 0.07	0.88 ± 0.05	0.53 ± 0.04	0.46 ± 0.04	0.3 ± 0.03	0.18 ± 0.02	0.12 ± 0.02	0.1 ± 0.01	0.16 ± 0.03	0.11 ± 0.02
Education										
Less than secondary	1.09 ± 0.1	1.23 ± 0.05	0.46 ± 0.06	0.6 ± 0.04	0.44 ± 0.06	0.33 ± 0.03	0.06 ± 0.02	0.07 ± 0.01	0.09 ± 0.03	0.19 ± 0.02
Secondary	1.33 ± 0.06 ^c^	1.28 ± 0.05	0.61 ± 0.04 ^c^	0.52 ± 0.03	0.47 ± 0.03	0.34 ± 0.02	0.11 ± 0.02	0.16 ± 0.02 ^c^	0.12 ± 0.02	0.21 ± 0.03
Some post-secondary	1.54 ± 0.08 ^c^	1.33 ± 0.06	0.67 ± 0.05 ^c^	0.57 ± 0.03	0.53 ± 0.04	0.4 ± 0.03	0.15 ± 0.02 ^c^	0.15 ± 0.02 ^c^	0.15 ± 0.03	0.16 ± 0.03
Post-secondary	1.41 ± 0.06 ^c^	1.32 ± 0.05	0.62 ± 0.04 ^c^	0.58 ± 0.03	0.41 ± 0.03	0.33 ± 0.03	0.18 ± 0.02 ^c^	0.19 ± 0.02 ^c^	0.17 ± 0.03 ^c^	0.18 ± 0.03
Income (CAD$/y)										
<50,000	1.46 ± 0.1	1.26 ± 0.04	0.71 ± 0.07	0.57 ± 0.03	0.45 ± 0.05	0.33 ± 0.02	0.12 ± 0.02	0.14 ± 0.01	0.14 ± 0.03	0.17 ± 0.02
50,000–100,000	1.33 ± 0.05	1.31 ± 0.05	0.58 ± 0.03	0.52 ± 0.02	0.43 ± 0.03	0.39 ± 0.03	0.14 ± 0.02	0.15 ± 0.01	0.15 ± 0.02	0.2 ± 0.03
100,000–150,000	1.49 ± 0.08	1.37 ± 0.07	0.65 ± 0.05	0.61 ± 0.05	0.51 ± 0.04	0.35 ± 0.03	0.16 ± 0.02	0.16 ± 0.03	0.14 ± 0.03	0.2 ± 0.04
>150,000	1.42 ± 0.08	1.33 ± 0.11	0.56 ± 0.04	0.6 ± 0.05	0.52 ± 0.06	0.36 ± 0.07	0.15 ± 0.02	0.16 ± 0.03	0.15 ± 0.04	0.18 ± 0.05
Province										
British Columbia	1.12 ± 0.08	1.23 ± 0.07	0.49 ± 0.04	0.5 ± 0.04	0.36 ± 0.05	0.31 ± 0.03	0.12 ± 0.03	0.22 ± 0.03	0.02 ± 0.01	0.02 ± 0.01
Alberta	1.49 ± 0.08 ^d^	1.34 ± 0.011	0.6 ± 0.05	0.62 ± 0.06	0.59 ± 0.06 ^d^	0.3 ± 0.03	0.1 ± 0.03	0.22 ± 0.05	0.02 ± 0.01	0.05 ± 0.03
Saskatchewan	1.31 ± 0.13	1.52 ± 0.011 ^d^	0.61 ± 0.09	0.69 ± 0.05 ^d^	0.42 ± 0.06	0.39 ± 0.05	0.12 ± 0.03	0.26 ± 0.06 ^d^	0.03 ± 0.01	0.06 ± 0.02
Manitoba	1.41 ± 0.11 ^d^	1.45 ± 0.14	0.69 ± 0.07	0.6 ± 0.05	0.5 ± 0.06	0.39 ± 0.07	0.15 ± 0.05	0.23 ± 0.05	0.04 ± 0.03	0.03 ± 0.02
Ontario	1.36 ± 0.08 ^d^	1.2 ± 0.05	0.62 ± 0.04 ^d^	0.55 ± 0.03	0.43 ± 0.04	0.33 ± 0.03	0.18 ± 0.03	0.17 ± 0.03 ^d^	0.02 ± 0.01	0.03 ± 0.01
Quebec	1.67 ± 0.09 ^d^	1.41 ± 0,05 ^d^	0.74 ± 0.07 ^d^	0.57 ± 0.03	0.53 ± 0.05 ^d^	0.44 ± 0.03 ^d^	0.12 ± 0.03 ^d^	0.15 ± 0.03	0.02 ± 0.01	0.04 ± 0.01
Atlantic provinces	1.42 ± 0.06 ^d^	1.31 ± 0.05	0.63 ± 0.04 ^d^	0.59 ± 0.03 ^d^	0.47 ± 0.03	0.32 ± 0.02	0.18 ± 0.03	0.21 ± 0.02	0.02 ± 0.01	0.03 ± 0.01
Reporter Status										
Under-reporters	0.82 ± 0.06 ^e^	0.81 ± 0.04 ^e^	0.35 ± 0.03 ^e^	0.44 ± 0.03 ^e^	0.26 ± 0.02 ^e^	0.2 ± 0.02 ^e^	0.11 ± 0.01 ^e^	0.09 ± 0.02 ^e^	0.09 ± 0.02	0.2 ± 0.02 ^e^
Plausible-reporters	1.59 ± 0.07	1.48 ± 0.03	0.7 ± 0.04	0.66 ± 0.03	0.52 ± 0.04	0.38 ± 0.02	0.17 ± 0.02	0.18 ± 0.02	0.15 ± 0.02	0.06 ± 0.01
Over-reporters	2.64 ± 0.17 ^e^	2.34 ± 0.19 ^e^	1.11 ± 0.14 ^e^	0.68 ± 0.06	1.09 ± 0.13 ^e^	0.99 ± 0.14 ^e^	0.17 ± 0.04	0.23 ± 0.04	0.22 ± 0.06	0.36 ± 0.08

^a^*p* < 0.05, significantly different from estimate for females within same age group. ^b^ *p* < 0.05, significantly different from estimate for non-Caucasians within the same age group. ^c^ *p* < 0.05, significantly different from estimate for those with less than secondary education within the same age group. ^d^ *p* < 0.05, significantly different from estimate for residents of British Columbia. ^e^ *p* < 0.05, significantly different from estimate for plausible-reporters.

**Table 3:** Percentage contribution of nutrients and energy from milk and alternatives and mean intakes based on 1-d intakes from the 2015 Canadian Community Health Survey—Nutrition (*n* = 13,616).

Rank	Nutrient/Energy	Contribution (%) ±SE	Per capita Mean 1-d Intake ±SE
1	Calcium (mg)	52.62 ± 0.46	411.61 ± 7.41
2	Vitamin D (mcg)	38.53 ± 0.78	1.82 ± 0.04
3	Saturated fat (g)	28.84 ± 0.51	6.55 ± 0.15
4	Vitamin B12 (mcg)	27.73 ± 0.57	1.13 ± 0.02
5	Vitamin A (mcg)	26.16 ± 0.58	170.43 ± 3.15
6	Phosphorus (mg)	24.76 ± 0.35	316.45 ± 5.68
7	Riboflavin (mg)	24.43 ± 0.37	0.47 ± 0.01
8	Protein (g)	16.30 ± 0.28	12.96 ± 0.24
9	Sugar (g)	16.22 ± 0.32	14.09 ± 0.3
10	Zinc (mg)	15.63 ± 0.27	1.65 ± 0.03
11	Total fat (g)	15.49 ± 0.32	10.83 ± 0.25
12	Cholesterol (mg)	14.22 ± 0.35	38.73 ± 0.84
13	Potassium (mg)	12.58 ± 0.22	337.91 ± 6.39
14	Sodium (mg)	12.15 ± 0.25	332.31 ± 7.71
15	Energy (kcal)	11.25 ± 0.19	211.3 ± 4.02
16	Monounsaturated fat (g)	11.10 ± 0.25	61.9 ± 0.07
17	Magnesium (mg)	9.65 ± 0.17	29.7 ± 0.54
18	Niacin (mg)	8.61 ± 0.18	3.38 ± 0.07
19	Carbohydrates (g)	7.00 ± 0.14	15.58 ± 0.33
20	Linolenic acid (g)	5.69 ± 0.16	0.09 ± 0.003
21	Vitamin B6 (mg)	5.53 ± 0.11	0.09 ± 0.002
22	Thiamine (mg)	4.98 ± 0.11	0.08 ± 0.002
23	Folate (mcg)	3.48 ± 0.07	15.35 ± 0.3
24	Polyunsaturated fat (g)	3.28 ± 0.09	0.49 ± 0.01
25	Linoleic acid (g)	2.92 ± 0.08	0.37 ± 0.01
26	Iron (mg)	1.67 ± 0.04	0.21 ± 0.01
27	Fibre (g)	1.03 ± 0.07	0.18 ± 0.01
28	Vitamin C (mg)	0.67 ± 0.09	0.66 ± 0.08

Nutrient intakes are those reported from food sources alone and do not account for those from supplements. Missing values for vitamin A (*n* = 5; 0.03%), sugar (*n* = 20; 0.1%), total fat (*n* = 5; 0.03%), cholesterol (*n* = 5; 0.03%), potassium (*n* = 5; 0.03%), sodium (*n* = 5; 0.03%), monounsaturated fat (*n* = 5; 0.03%), niacin (*n* = 25; 0.13%), linolenic acid (*n* = 48; 0.25%), vitamin B6 (*n* = 25; 0.13%), thiamine (*n* = 24; 0.13%), folate (*n* = 5; 0.03%), polyunsaturated fat (*n* = 5; 0.03%), linoleic acid (*n* = 48; 0.25%), iron (*n* = 5; 0.03%), fibre (*n* = 60; 0.31%), and vitamin C (*n* = 5; 0.03%).

**Table 4:** Percentage contribution of nutrients and energy from milk and alternative sources and mean intakes per capita and per consumer based on 1-d intakes from the 2015 Canadian Community Health Survey—Nutrition (*n* = 13,616).

Nutrient or Energy	Top Source	Contribution (%) ±SE	Per capita Mean 1-d Intake ± SE	Per Consumer Mean 1-d Intake ± SE
Vitamin D (mcg)	Milk	33 ± 0.74	1.56 ± 0.04	2.04 ± 0.05
Calcium (mg)	Milk	24.20 ± 0.47	189.30 ± 4.66	247.64 ± 5.62
Vitamin B12 (mcg)	Milk	18.06 ± 0.46	0.74 ± 0.02	0.96 ± 0.02
Saturated fat (g)	Cheese	16.53 ± 0.46	3.75 ± 0.12	6.86 ± 0.2
Riboflavin (mg)	Milk	14.88 ± 0.32	0.29 ± 0.01	0.37 ± 0.01
Vitamin A (mcg)	Milk	14.55 ± 0.39	94.80 ± 2.24	124.01 ± 2.68
Phosphorus (mg)	Milk	11.47 ± 0.25	146.59 ± 3.6	191.76 ± 4.36
Sugar (g)	Milk	9.59 ± 0.22	8.33 ± 0.2	10.9 ± 0.25
Total fat (g)	Cheese	8.88 ± 0.27	6.21 ± 0.21	11.34 ± 0.33
Potassium (mg)	Milk	8.46 ± 0.2	227.4 ± 5.61	297.48 ± 6.78
Sodium (mg)	Cheese	8.44 ± 0.24	230.67 ± 7.2	421.37 ± 11.15
Cholesterol (mg)	Cheese	7.62 ± 0.25	20.76 ± 0.66	37.93 ± 1.06
Protein (g)	Cheese	7.33 ± 0.21	5.82 ± 0.17	10.64 ± 0.27
Zinc (mg)	Cheese	7.12 ± 0.21	0.75 ± 0.02	1.37 ± 0.04
Monounsaturated fat (g)	Cheese	6.19 ± 0.2	1.62 ± 0.05	2.96 ± 0.09
Magnesium (mg)	Milk	5.63 ± 0.14	17.35 ± 0.43	22.69 ± 0.52
Energy (kcal)	Cheese	4.49 ± 0.13	84.36 ± 2.65	154.1 ± 4.16
Niacin (mg)	Milk	4.27 ± 0.14	1.26 ± 0.03	1.65 ± 0.04
Carbohydrates (mg)	Milk	3.70 ± 0.09	10.95 ± 0.33	14.32 ± 0.4
Thiamine (mg)	Milk	3.56 ± 0.09	0.06 ± 0.002	0.07 ± 0.002
Vitamin B6 (mg)	Milk	3.41 ± 0.09	0.06 ± 0.002	0.08 ± 0.002
Linolenic acid (mg)	Cheese	2.55 ± 0.08	0.04 ± 0.001	0.07 ± 0.003
Folate (mcg)	Milk	1.77 ± 0.05	7.79 ± 0.2	10.19 ± 0.23
Polyunsaturated fat (g)	Cheese	1.59 ± 0.06	0.24 ± 0.01	0.43 ± 0.01
Linoleic acid (g)	Cheese	1.45 ± 0.06	0.18 ± 0.01	0.33 ± 0.01
Iron (mg)	Cheese	0.58 ± 0.02	0.07 ± 0.002	0.13 ± 0.003
Fibre (g)	Frozen dairy	0.53 ± 0.06	0.09 ± 0.01	0.82 ± 0.09
Vitamin C (mg)	Milk	0.41 ± 0.08	0.4 ± 0.08	0.53 ± 0.11

Nutrient intakes are those reported from food sources alone and do not account for those from supplements. Missing values for vitamin D (*n* = 12; 0.07%), calcium (*n* = 12; 0.07%), vitamin B12 (*n* = 1; 0.006%), riboflavin (*n* = 1; 0.006%), vitamin A (*n* = 1; 0.006%), phosphorus (*n* = 1; 0.006%), sugar (*n* = 12; 0.07%), potassium (*n* = 12; 0.07%), magnesium (*n* = 1; 0.006%), niacin (*n* = 1; 0.006%), carbohydrates (*n* = 1; 0.006%), thiamine (*n* = 12; 0.07%), vitamin B6 (*n* = 1; 0.006%), folate (*n* = 1; 0.006%), and vitamin C (*n* = 1; 0.006%) from milk. Missing values for total fat (*n* = 130; 1.13%), sodium (*n* = 3; 0.03%), cholesterol (*n* = 3; 0.03%), zinc (*n* = 3; 0.03%), energy (*n* = 848; 7.86%), linolenic acid (*n* = 3; 0.03%), polyunsaturated fat (*n* = 3; 0.03%), linoleic acid (*n* = 3; 0.03%), and iron (*n* = 3; 0.03%) from cheese.

## Appendix A

**Table A1:** Food classifications and codes used to classify milk and alternatives.

Food Source	Food Classification	Food Codes and Descriptions
Milk and alternatives	CNF/CFG	3101, 3102, 3103, 3104: fluid milk and fortified soy-based beverages; 3201, 3202, 3203, 3204: other milk and alternatives (cheese, yoghurt)
Cheese	BNS	14A: cottage cheese; 14B: cheese, less than 10% B.F.; 14C: cheese, 10% B.F. to 25% B.F.; 14D: cheese, more than 25% B.F.
Milk	BNS	10A: milk, whole; 10B: milk, 2%; 10C: milk, 1%; 10D: milk, skim; 10E: milk, evaporated, whole; 10F: milk, evaporated, 2%; 10G: milk, evaporated, skim; 10H: milk, condensed; 10I: other types of milk (whey, buttermilk); 10K: goat and sheep milk
Yoghurt	BNS	15A: yoghurts, less than 2% B.F.; 15B: yoghurts, more than 2.1% B.F.
Frozen dairy	BNS	09A: ice cream; 09B: ice milk; 09C: frozen yoghurt
Other dairy	BNS	08B: cakes, commercial (frozen cake); 18A: regular tub margarine; 231D: milk-based beverages (milk shakes, malted milk, hot cocoa, instant breakfast, etc.); 43C: Jell-O, dessert toppings and pudding mixes, commercial; 46D: other beverages (malted milk, chocolate beverage); 50A: soups with vegetables; 50B soups without vegetables; 50D: sauces (white, bearnaise, soya, tartar, ketchup, etc.)
Soy products	NSS	5241: plant-based beverage, soy, original and vanilla, unenriched; 5429: plant-based beverage, soy, unenriched, chocolate; 6329: plant-based beverage, soy, enriched, chocolate; 6330: plant-based beverage, soy, enriched, all flavours, unsweetened; 6332: plant-based beverage, soy, enriched, all flavours, fat free; 6666: plant-based beverage, soy, enriched, with omega-3 fatty acids added; 6720: plant-based beverage, soy, enriched, all flavours; 6784: plant-based beverage, soy, enriched, all flavours, reduced fat; 404,054: cheese, soy, slices; 404,064: soy-based beverage, powder; 504,723: pudding, all flavours, prepared with soy beverage

Abbreviations: CNF/CFG, Canadian Nutrient File/Canada’s Food Guide classification; B.F.: butter fat; BNS, Bureau of Nutritional Sciences; NSS, Nutrition Survey System.
